# 4-aminopyridine attenuates inflammation and apoptosis and increases angiogenesis to promote skin regeneration following a burn injury

**DOI:** 10.21203/rs.3.rs-4669610/v1

**Published:** 2024-08-01

**Authors:** Prem Kumar Govindappa, Rahul V.G, Govindaraj Ellur, Amir A. Gaber, John Elfar

**Affiliations:** The University of Arizona College of Medicine; The University of Arizona College of Medicine; The University of Arizona College of Medicine; The University of Arizona College of Medicine; The University of Arizona

## Abstract

Severe thermal skin burns are complicated by inflammation and apoptosis, which delays wound healing and contributes to significant morbidity. Diverse treatments demonstrate limited success with mitigating these processes to accelerate healing. Agents that alter cell behavior to improve healing would alter treatment paradigms. We repurposed 4-aminopyridine (4-AP), a drug approved by the US FDA for multiple sclerosis, to treat severe burns. We found that 4-AP, in the early stages of burn healing, significantly reduced the expression of pro-inflammatory cytokines IL1β and TNFα while increasing the expression of anti-inflammatory markers CD206, ARG-1, and IL10. 4-AP attenuated apoptosis, with decreases in apoptotic markers BAX, caspase-9, and caspase-3 and increases in anti-apoptotic markers BCL2 and BCL-XL. Furthermore, 4-AP promoted angiogenesis through increases in the expression of CD31, VEGF, and eNOS. Together, these likely contributed to accelerated burn wound closure, as demonstrated in increased keratinocyte proliferation (K14) and differentiation (K10) markers. In the later stages of burn healing, 4-AP increased TGFβ and FGF levels, which are known to mark the transformation of fibroblasts to myofibroblasts. This was further demonstrated by an increased expression of α-SMA and vimentin, as well as higher levels of collagen I and III, MMP 3, and 9 in animals treated with 4-AP. Our findings support the idea that 4-AP may have a novel, clinically relevant therapeutic use in promoting burn wound healing.

## INTRODUCTION

Skin burns most often result from exposure to hot solids and liquids, flames, chemicals, radiation, and other sources of heat^1^. The American Burn Association (ABA) reports that approximately 486,000 patients suffer from burn injuries annually, resulting in a total cost of $ 42.4 billion^2,3^. Thermal burns account for about 86% of burns, with direct solid-object contact causing 9% of these burns^4^. The pathophysiological changes caused by heated burns are complex and vary depending on burn type and severity^5^. Severe burns (third degree) damage the skin’s structural integrity, leading to increased inflammation, apoptosis, and reduced oxygen supply with inadequate tissue perfusion^6^.

The immune response to a severe burn injury comprises the initial proinflammatory reaction, which is followed by an anti-inflammatory resolution phase. These phases determine the size and severity of the wound^7^. Macrophages are primary immune cells of the skin and they play a vital role in burn injury via the transformation from M1 (pro-inflammatory) to M2 (reparative) phenotypes, which improves angiogenesis and helps to control inflammation – a key driver of local cell death and burn expansion^8^. Inefficient control of inflammation is the primary driver of secondary burn-wound progression, impaired tissue regeneration and delayed healing^9^. Several studies demonstrated that depletion of dermal macrophages during wound healing has a detrimental impact on wound closure. These macrophage deficient or depleted models feature decreased vessel density, decreased fibroblast to myofibroblast differentiation, and delayed re-epithelization^10–13^. Few current treatments address these processes and much of current burn care is focused on metabolic and fluid support, as well as infection control^14^. Ideal therapies would increase tissue protection by protecting burned tissue from the effects of inflammation and cell death to bolster oxygenation. This would improve healing, decrease secondary burn progression and reduce the morbidity and mortality associated with severe burns.

4-aminopyridine (4-AP), a potassium channel blocker and calcium channel agonist, is an FDA-approved treatment used for multiple sclerosis and chronic neurodegenerative conditions^15^. We recently found that 4-AP treatment enhanced wound healing and hair follicle regeneration by augmenting keratinocyte, fibroblast, and Schwann-cell function in a splinted full-thickness skin wound model^16^. In addition to pursuing an approved clinical trial in human wounds^17^, these findings motivated us to study the effects of 4-AP in a standard burn model where we could study 4-AP’s effects on secondary burn progression. We hypothesized that 4-AP attenuates early inflammation and apoptosis, possibly through accelerated angiogenesis, which helps to prevent the secondary burn progression to accelerate burn closure. We therefore examined 4-AP’s effect on inflammation, apoptosis, angiogenesis, extracellular matrix (ECM) remodeling, as well as cellular phenotypic changes in keratinocytes, macrophages and fibroblasts.

We found that 4-AP reduced early inflammation and apoptosis and promoted angiogenesis to expedite burn closure, ECM regeneration, and remodeling. Our data supports a novel therapeutic effect use for 4-AP in promoting cutaneous burn healing, which is clinically translatable.

## RESULTS

### 4-AP expedited burn injury closure and enhanced skin regeneration following skin burn

**Figure 1:**
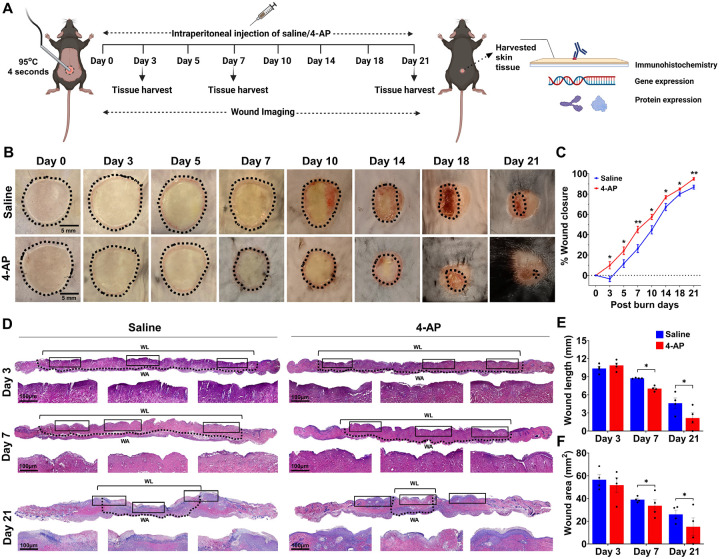
4-AP expedited burn injury closure and enhanced skin regeneration. **A)** Schematic illustration of experimental design to test the beneficial therapeutic effect of 4-AP in the C57BL/6 mouse burned model. **B)** Representative digital images of burn healing in saline and 4-AP (2 mg/kg/IP, single dose immediately after surgery and until day 21) treated mice at 0, 3, 5, 7, 10, 14, 18, and 21 days post-burning. Scale bar = 5 mm. **C)** Percent burn wound closure at each time point relative to the initial burn wound area in control and 4-AP-treated mice. n = 6 animals per group, with one burn wound per animal. **D)**Representative images of H&E-stained sections of full-thickness burn of saline and 4-AP treated skin tissue on days 3, 7, and 21. Scale bar = 100 μm, n =4 skin tissues per group. **E)** Quantification of burn wound length and area in H&E-stained skin burn sections using ImageJ software on days 3, 7, and 21. n = 4 skin tissues per group. Data are represented as mean ± SEM. The statistical significance is indicated by asterisks (*P < 0.05, and **P < 0.0021 vs. saline group) and compared using two-tailed, unpaired t-tests.

Mouse burn models are commonly used to evaluate tissue regeneration and repair^18^. To test the hypothesis that systemic 4-AP treatment promotes burn wound closure and healing, we created a 10-mm-diameter heat-induced, severe, full-thickness skin burn on the dorsal thoracolumbar surface of mice. Approved burn model experimental details are shown in Fig. 1A. Macroscopic imaging burns (Fig. 1B) allowed the assessment of burn closure percentage over time (Fig. 1C; *P < 0.05 and **P < 0.0021) revealed accelerated burn closure in 4-AP-treated mice compared to saline controls. Accelerated healing was attributable to early protection against secondary expansion of burn injury with 4-AP treatment, evidenced as the difference at day 3 post burn injury where 4-AP treated animals closed 9.87% of their burn area, whereas saline treated animals expanded their wounds an average of 3.39% (average % wound closure difference was 13.26%, *P < 0.05). Thereafter, burn closure in the 4-AP group consistently outpaced saline controls on day 5 (24.24 vs. 11.61), day 7 (45.09 vs. 26.38), day 10 (57.48 vs. 44.76), day 14 (76.86 vs. 67.17), day 18 (84.92 vs. 80.06), and day 21 (94.96 vs. 86.80). By day 21, 4-AP treated animals had all healed, while the saline-treated animals had not. The dermis area and dermal length (distance between healing epidermal tongues) were measured using H&E histology (Fig. 1D). Quantitative analysis of burn wound area (Fig. 1F; *P < 0.05) on day 7 (33.62 vs. 39.03) and day 21 (15.14 vs. 26.11) and burn wound length (Fig. 1E; *P < 0.05) on the same days (7.03 vs. 8.75 and 2.15 vs. 4.64) confirmed that 4-AP accelerates tissue healing compared to saline. Burn wound area benefits manifested after day 3. Compared to saline, 4-AP treatment resulted in significant differences in the structural organization of the epidermis and dermis, which was likely attributable to the proliferation, migration, and transformation of keratinocytes, immune cells, and fibroblasts (Fig. 1D; magnified area). There was a considerable effect on accelerating granulation, re-epithelialization, and burn wound-healing closure rate in 4-AP treated animals.

### 4-AP attenuated inflammation and augmented anti-inflammatory effects following skin burn

**Figure 2:**
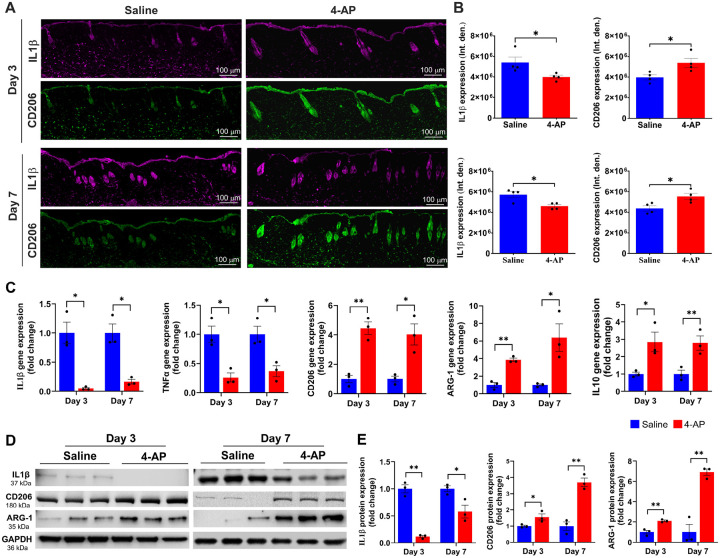
4-AP attenuated pro-inflammation and accelerated anti-inflammatory effects following skin burn. **A and B)** Representative images of IF staining of IL1β (pro-inflammatory) and CD206 (anti-inflammatory) and its quantitative results where 4-AP significantly controlled inflammatory resolution compared to saline-treated skin burn tissues on days 3 and 7. Scale bar = 100 μm. n = 4 skin tissues per group. **C)** qRT-PCR data shows that 4-AP treatment significantly attenuated pro-inflammatory genes (IL1β and TNFα; M1 macrophage markers) and upregulated anti-inflammatory genes (CD206, ARG-1, and IL10; M2 macrophage markers) compared to the saline group on days 3 and 7. n = 3 skin tissues per group. **D and E)** Western blotting images and quantitative results show that 4-AP treatment significantly attenuated IL1β and upregulated CD206 and ARG-1 protein expressions compared to the saline group on days 3 and 7. n = 3 skin tissues per group. Data are represented as mean ± SEM, and the statistical significance is indicated by asterisks (*P < 0.05 and **P < 0.0021 vs. saline group) and compared using two-tailed, unpaired t-tests.

We aimed to test 4-AP’s effect on post-burn inflammation, which is the earliest response to burn injury, lasting for weeks depending on burn severity^19^. Inflammation causes keratinocytes and fibroblasts release factors that attract inflammatory macrophages and stimulate angiogenesis^20^. The quantitative results of the macrophage IF analysis showed that 4-AP treatment significantly shifted macrophage polarization from the M1 (IL1β; Inflammatory) to M2 (CD206; Reparative) phenotype in tissues abutting the burn site on days 3, and 7 (Fig. 2A, B; *P < 0.05). These data suggest a role for 4-AP in activation and recruitment of macrophages. 4-AP treatment also significantly decreased pro-inflammatory genes (IL1β and TNFα), and increased the expression of anti-inflammatory genes (CD206, ARG-1, and IL10) compared to the saline group (Fig. 2C; *P < 0.05 and **P < 0.0021). These changes were also evident at the protein expression level, with 4-AP significantly attenuating IL1β expression, while increasing expression of CD206, and ARG-1 proteins compared with saline treatment (Fig. 2D, E; *P < 0.05 and **P < 0.0021). Taken together, these results suggest a key role for 4-AP treatment in modulating inflammatory resolution in skin burns, with possible beneficial effects on early angiogenesis (Fig. 8A, C. Schematic illustration).

### 4-AP augmented angiogenesis following skin burn

**Figure 3:**
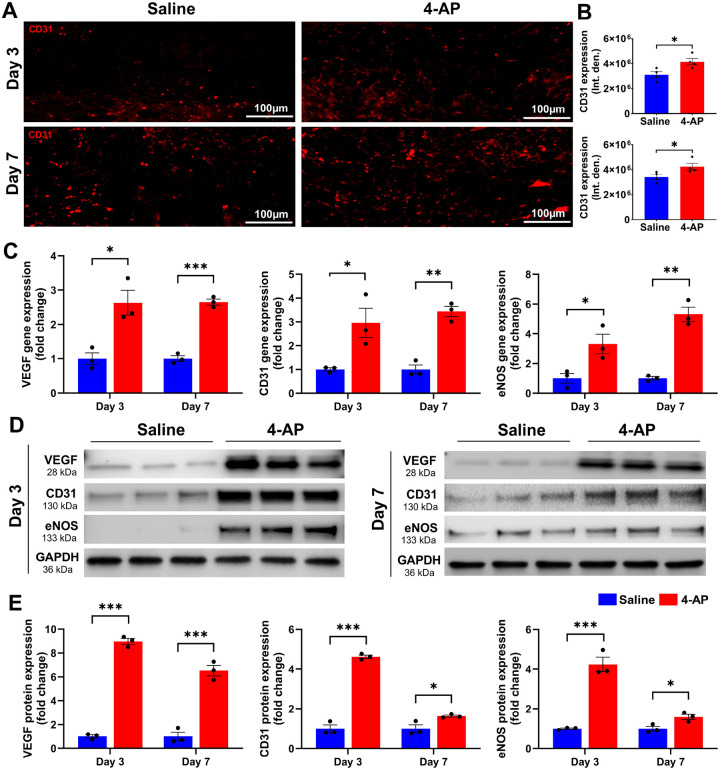
4-AP enhanced angiogenesis following skin burn. **A and B)** Representative images and quantitative results of IF staining of CD31 in saline and 4-AP treated skin burn tissues on days 3 and 7. Scale bar = 100 μm. n = 4 skin tissues per group. **C)** qRT-PCR data shows that 4-AP treatment significantly increased the expression of angiogenesis genes (VEGF, CD31, and eNOS) compared to the saline group on days 3 and 7. n = 3 skin tissues per group. **D and E)** Western blotting images and quantitative results showed that 4-AP treatment significantly increased angiogenesis protein expressions (VEGF, CD31, and eNOS) compared to the saline group on days 3 and 7. n = 3 skin tissues per group. Data are represented as mean ± SEM. The statistical significance is indicated by asterisks (*P < 0.05, **P < 0.0021, and ***P < 0.0002 vs. saline group) and compared using two-tailed, unpaired t-tests.

**Figure 4:**
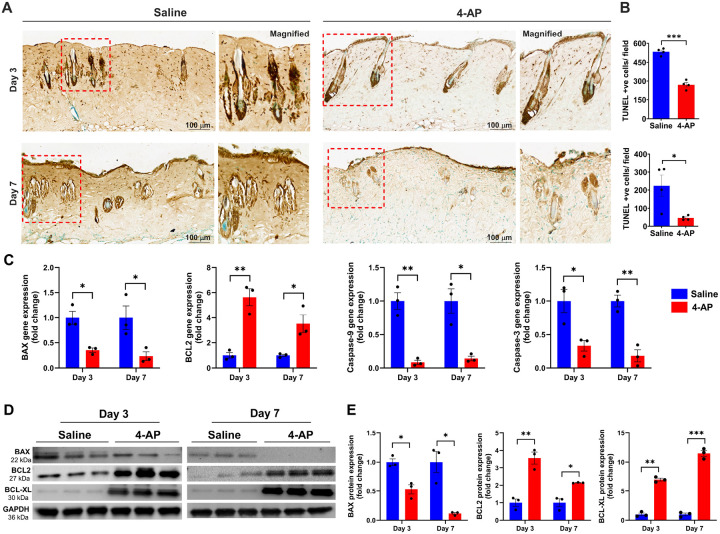
4-AP attenuated pro-apoptosis and accelerated anti-apoptosis effects following skin burn. **A and B)** Representative images of DAB-TUNEL staining of apoptosis and quantitative results of TUNEL positive cells in saline and 4-AP treated skin burn tissues on days 3 and 7. n = 4 skin tissues per group. **C)**qRT-PCR data shows that 4-AP treatment significantly attenuated pro-apoptosis genes (BAX, caspase-9, and caspase-3) and upregulated anti-apoptosis genes (BCL2) compared to the saline group on days 3 and 7. n = 3 skin tissues per group. **D and E)** Western blotting images and quantitative results showed that 4-AP treatment significantly attenuated expressions of pro-apoptosis protein (BAX) and upregulated anti-apoptosis proteins (BCL2 and BCL-XL) as compared to the saline group on days 3 and 7. n = 3 skin tissues per group. Data are represented as mean ± SEM. The statistical significance is indicated by asterisks (*P < 0.05, **P < 0.0021, and ***P < 0.0002 vs. saline group) and compared using two-tailed, unpaired t-tests.

Revascularization through neo angiogenesis begins immediately after burns and provides vital oxygen and nutrients for healing^23^. Impaired angiogenesis impedes burn wound regeneration^24^, and we tested the hypothesis that 4-AP treatment could enhance angiogenesis early after burns. We found increased CD31 IF staining at days 3 and 7 post burn with 4-AP treatment compared to saline (Fig. 3A, B; *P < 0.05). Corresponding changes were found in the gene and protein expression level of several known markers of neo-angiogenesis, including VEGF, CD31, and eNOS with 4-AP treatment (gene expression, Fig. 3C; *P < 0.05, **P < 0.0021, and ***P < 0.0002; corresponding protein expression Fig. 3D, E; *P < 0.05 and ***P < 0.0002) as compared to the saline group. Histomorphometric evaluation of tissue specimens confirmed effects on vascular augmentation with 4-AP treatment (Fig. 8C. Schematic illustration) that is considered crucial to both regeneration and the control of post-burn inflammation (Fig. 2) and as well as apoptosis (Fig. 4).

### 4-AP attenuated apoptosis and increased anti-apoptosis markers following skin burn

Recent studies showed that burns cause significant apoptosis or cell death, which hinders skin cell regenerative efforts and impedes healing^21,22^. To investigate the anti-apoptotic effect of 4-AP after a burn injury, we used DAB-TUNEL to stain burned tissues (Fig. 4A). On day 3, 4-AP treatment significantly reduced apoptosis compared to saline treatment (270 vs. 533 per field; Fig. 4A, B; ***P < 0.0001). By day 7, 4-AP treatment continued to show protective effects (vs. day 3, 4-AP treatment) against apoptosis (47 vs. 270 per field; Fig. 4A, B; *P < 0.05) compared to saline treatment (47 vs. 224 per field; Fig. 4A, B; *P < 0.05). On days 3 and 7 post injury, saline-treated mice showed noticeable apoptosis in the skin hair follicles and the epidermis (keratinocytes) and dermis (fibroblasts) regions (Fig. 4A; magnified field), whereas 4-AP treatment resulted in significantly reduced apoptosis and increased regeneration of new cells in these regions and in hair follicle bulges (green cells). Treatment with 4-AP attenuated the gene expression of pro-apoptosis (BAX, caspase-9, and caspase-3) and bolstered the expression of anti-apoptosis (BCL2) (Fig. 4C; *P < 0.05 and **P < 0.0021) and this also led to corresponding changes at protein levels (Fig. 4D, E; *P < 0.05, **P < 0.0021, and ***P < 0.0002) on days 3 and 7 post-burn injury. This significant positive effect of 4-AP on preventing apoptosis and augmenting anti-apoptosis (Fig. 8B. Schematic illustration) may be the basis for the accelerating functional burn wound closure after burn injury (Fig. 1B–F).

### 4-AP accelerated re-epithelization following skin burn

**Figure 5:**
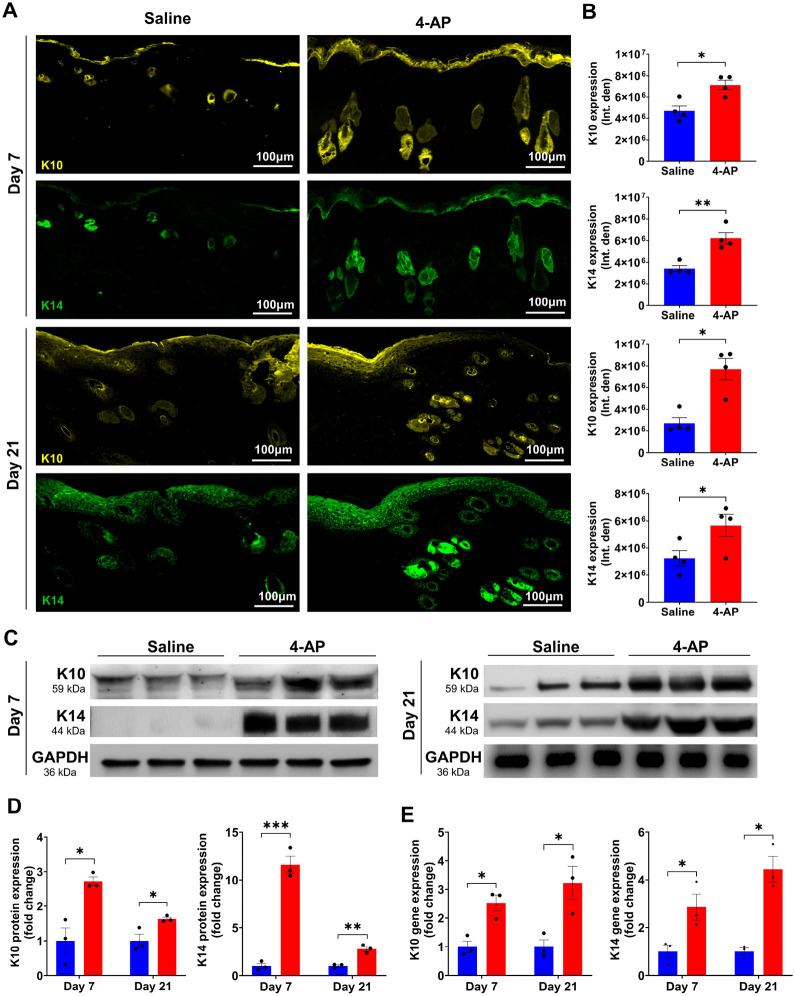
4-AP accelerated epithelization and keratinocytes terminal differentiation following skin burn. **A and B)** Representative images and quantitative results of IF staining of keratinocyte markers (K10; terminal differentiation and K14; proliferative) in saline and 4-AP treated skin burn tissues on days 3 and 7. Scale bar = 100 μm. n = 4 skin tissues per group. **C and D)** Western blotting and **E)** qRT-PCR quantitative results show that 4-AP treatment significantly increased keratin markers protein and gene (K10 and K14) expressions compared to the saline group on days 3 and 7. n = 3 skin tissues per group. Data are represented as mean ± SEM. The statistical significance is indicated by asterisks (*P < 0.05, **P < 0.0021, and ***P < 0.0002 vs. saline group) and compared using two-tailed, unpaired t-tests.

Re-epithelialization is crucial for providing a new barrier between deeper exposed tissues and the bacteria-laden outside environment^25^. Several studies show that keratin markers K10 (terminal differentiation), and K14 (undifferentiation) are expressed in both epidermis and regenerating hair follicles^26,27^. Given our observations that 4-AP increased the thickness of healing epidermal tongues after burns as well as improved dermal regeneration (Fig. 1D; magnified images), we sought to investigate whether these observations were linked to an increased expression of these same markers in keratinocytes and whether there were changes in epidermal differentiation. IF staining analysis of keratin markers showed that 4-AP treatment significantly increased the expression of K10, and K14 in both epidermis and hair follicles on days 7, and 21 compared to the saline group (Fig. 5A, B; *P < 0.05 and **P < 0.0021). There was no significant difference in the IF expression of these markers on day 3 (data not shown), as would be expected as skin healing follows wound inflammation. We focused on gene and protein expression of these markers in burned skin tissues on days 7 and 21 and found that 4-AP treatment significantly increased both gene (Fig. 5E; *P < 0.05) and protein (Fig. 5C, D; *P < 0.05 and ***P < 0.0002) expression of K10 and K14. This was highly suggestive of positive effects on re-epithelialization for 4-AP reminiscent of effects we observed in standard wound healing^16^ (Fig. 8D. Schematic illustration).

### 4-AP promoted fibroblast to myofibroblast transformation following skin burn

**Figure 6:**
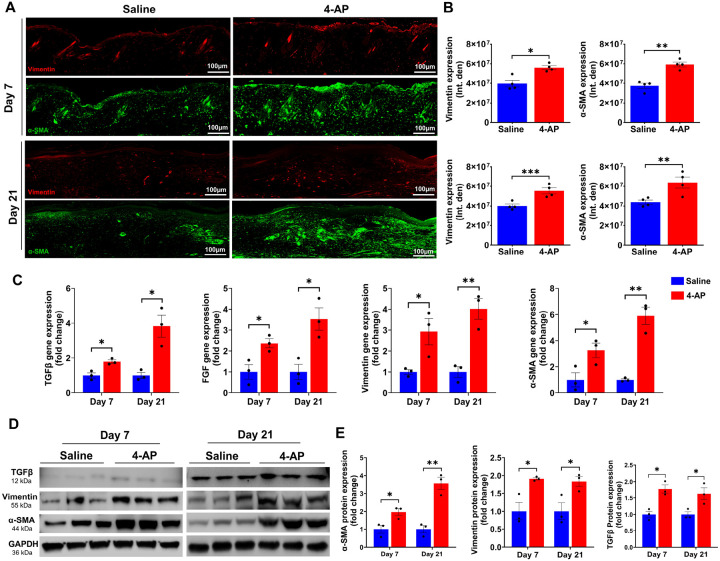
4-AP increased fibroblast to myofibroblast transformation following skin burn. **A and B)** Representative images and quantitative results of IF staining of vimentin (fibroblasts) and α-SMA (myofibroblasts) in saline and 4-AP treated skin burn tissues on days 3 and 7. Scale bar = 100 μm. n = 4 skin tissues per group**. C)** qRT-PCR data shows that 4-AP treatment significantly increased the expression of TGFβ, vimentin, α-SMA, and FGF genes compared to the saline group on days 7 and 21. n = 3 skin tissues per group. **D and E)** Western blotting images and quantitative results showed that 4-AP treatment significantly increased the expression of TGFβ, vimentin, and α-SMA proteins compared to the saline group on days 7 and 21. n = 3 skin tissues per group. Data are represented as mean ± SEM. The statistical significance is indicated by asterisks (*P < 0.05, **P < 0.0021, and ***P < 0.0002 vs. saline group) and compared using two-tailed, unpaired t-tests.

TGF-β plays a central role in the transition of fibroblasts (vimentin-positive cells) to myofibroblasts (α-SMA-positive cells), which is a crucial step in skin burn healing^28,29^. Fibroblasts, along with macrophages (immune cells), endothelial cells (vessels), and keratinocytes are all components of tissue granulation, where myofibroblasts play a crucial role in accelerating burn wound contraction, and ECM production^30^. Given our finding of 4-AP’s effects on the generation of M2 macrophages (Fig. 2), angiogenesis (Fig. 3), and keratinocytes (Fig. 5) in the burn wound tissues, we sought to investigate effects on the transformation of fibroblasts following burn injury on days 7 and 21. The results of IF staining showed that 4-AP significantly increased the expression of both vimentin and α-SMA positive cell types (Fig. 6A, B; *P < 0.05, **P < 0.0021, and ***P < 0.0002), which is evident on day 21. Next, we investigated the effects of 4-AP on vimentin, α-SMA, FGF, and TGFβ markers at the gene levels, where 4-AP notably increased the expression of all these gene markers (Fig. 6C; *P < 0.05 and **P < 0.0021) and protein expression (vimentin, α-SMA, and TGFβ proteins: Fig. 6D, E; *P < 0.05 and **P < 0.0021) compared with saline treatment. Our data supported a vital role for 4-AP treatment on TGFβ downstream signaling for burn tissue remodeling (Fig. 8E. Schematic illustration).

### 4-AP augmented matrix remodeling and tissue healing following skin burn

**Figure 7:**
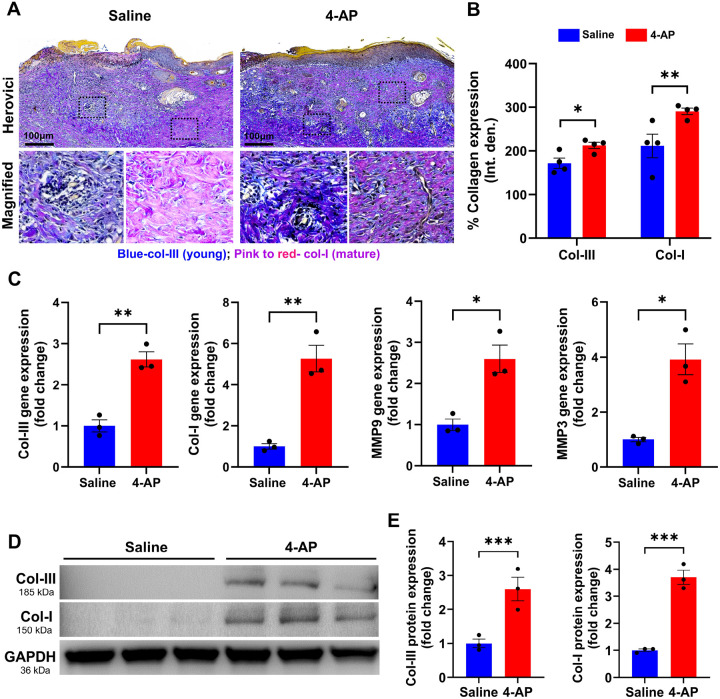
4-AP advanced skin burns wound repair by increasing collagen and MMP expressions. **A and B)** Representative images and quantitative results of Herovici staining of collagen III/I in saline and 4-AP treated skin burn tissues on days 7 and 21. n = 4 skin tissues per group. **C)** qRT-PCR data shows that 4-AP treatment significantly increased the expression of collagen-III/I and MM9/3 genes compared to the saline group on days 7 and 21. n = 3 skin tissues per group. **D and E)** Western blotting images and quantitative results showed that 4-AP treatment significantly increased collagen-III/I protein expressions compared to the saline group on day 21. n = 3 skin tissues per group. Data are represented as mean ± SEM. The statistical significance is indicated by asterisks (*P < 0.05, **P < 0.0021, and P*** < 0.0002 vs. saline group) and compared using two-tailed, unpaired t-tests.

**Figure 8:**
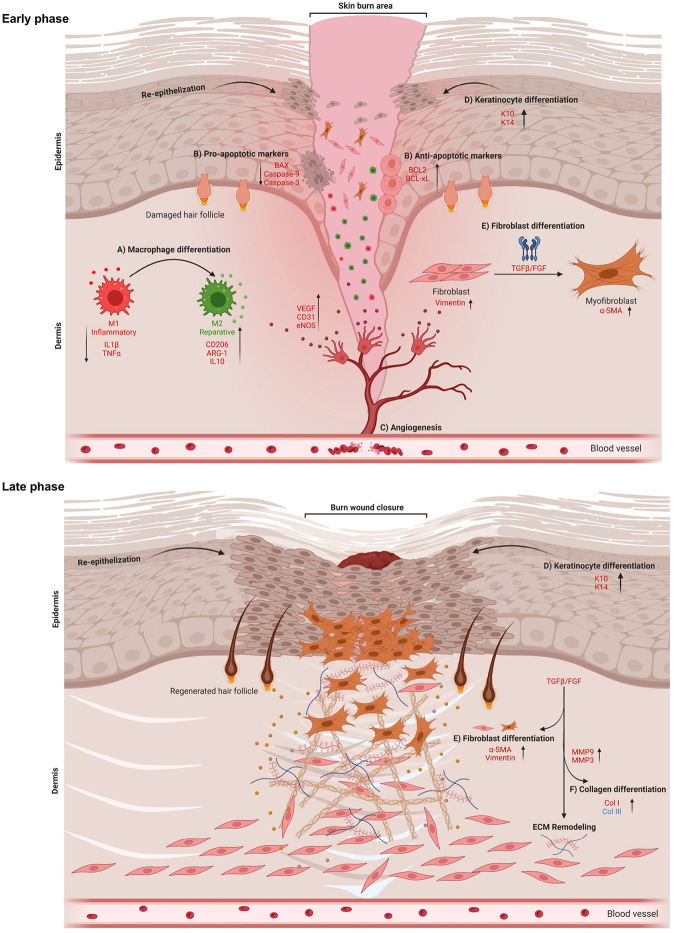
Schematic illustration of early and late phases of skin burn healing. 4-AP expedites burn wound healing via controlling multi-cellular events of **A)** macrophage, **B)** apoptosis, **C)** angiogenesis, **D)**keratinocyte, and **E)** fibroblast. During the early phase (days 3 and 7) of burn wound healing, 4-AP significantly mitigates inflammation and apoptosis and enhances angiogenesis, keratin, and fibroblast differentiation. In the later phase (day 21), 4-AP promotes wound closure by accelerating keratinocyte differentiation and accelerating the remodeling of the ECM via stimulating fibroblast and collagen differentiation.

Later in skin healing after a burn, fibroblasts, keratinocytes, and immune cells play a crucial role in remodeling the ECM^31^. These cells, especially fibroblasts, produce ECM proteins such as collagen types III and I and release matrix metalloproteinases (MMPs)^32^. MMPs such as MMP3 and MMP9 are responsible for synthesis and degradation of ECM, including collagen^33,34^. While healing, the production of type III collagen (early immature collagen product) increases, providing elasticity and resilience, while type I collagen (late mature collagen product) provides tensile strength^35,36^. We quantified the amounts of collagen I and III, as well as the expression of MMP9 and MMP3 on day 21. The results of the Herovici’s (collagen) staining showed that 4-AP significantly increased the expression of both collagen III and I (Fig. 7A, B; *P < 0.05 and **P < 0.0021). 4-AP’s effect on gene expression of collagen I, collagen III, MMP3 and MMP9 were all increased compared with saline (Fig. 7C; *P < 0.05 and **P < 0.0021), and this was true for protein expression of collagen I and III proteins (Fig. 7D, E; ***P < 0.0002) as well. This supports the idea that 4-AP had a pro-remodeling effect on ECM in healing burns (Fig. 8E. Schematic illustration).

## DISCUSSION

In previous work, we demonstrated that 4-AP accelerated wound healing in a full-thickness excisional mouse model by augmenting re-epithelialization, dermal regeneration, and reinnervation^16^. The effects we previously found were on multiple cell types, and were sufficient to satisfy the FDA requirements to pursue a trial through exemption on healthy patients with standard skin-punch biopsy wounds^17^. The effects on standard full-thickness wounds could not, however, be translated directly to an understanding of the 4-AP’s effects on thermal burns. Here we aimed to investigate, for the first time, whether 4-AP does indeed attenuate inflammation and apoptosis by enhancing angiogenesis in actual severe burns, and whether this would translate to accelerated burn wound closure. Our data showed that 4-AP treatment significantly attenuated inflammation and apoptosis, and enhanced angiogenesis and epidermal and dermal regeneration to accelerate burn wound closure.

After a burn injury, skin responses to the heat injury are critical to mitigating burn wound expansion and initiating tissue regeneration. We first focused on the impact of heat-induced burns on the skin, specifically looking at pro-inflammatory and macrophage responses and the role of 4-AP in controlling these factors. Burn injuries primarily cause inflammation, which can worsen if M1 macrophage phenotypes dominate over M2 macrophage populations^8,37^. This exacerbates inflammation and leads to cell death in the burn wounds^38^. The timely transformation of M1 to M2 macrophages is crucial for reducing inflammation, and supporting the release of growth factors to bolster angiogenesis^39^. We found 4-AP significantly reduced proinflammatory cytokines like IL-1β and TNFα while increasing anti-inflammatory cytokines like IL-10, ARG-1, and CD206 on days 3 and 7 after the burn. Interestingly, several studies suggest that depletion of macrophages results in defective wound repair^10–13,40^, likely through the release cytokines and growth factors, such as epidermal growth factor (EGF), keratinocyte growth factor (KGF), transforming growth factor-β (TGFβ), vascular endothelial growth factor (VEGF), and others^41,42^. These factors activate the proliferation and maturation of different cell types, especially keratinocytes, fibroblasts, and endothelial cells. This complex transformation contributes to burn wound granulation, re-epithelialization, and ECM repair. Our data suggests that 4-AP may accelerate burn wound closure by accelerating these transitions.

Burn injury-induced ischemia drives angiogenesis, which plays a critical role in tissue repair and regeneration^48^ by providing nutrients and oxygenation to ischemic tissues^49^ as well as facilitating the ingress of inflammatory neutrophils and macrophages to granulate the wound bed^50^. Unsuccessful revascularization of burned tissues impedes the resolution of inflammation in burned tissue^51^. VEGF is a potent mediator of angiogenesis and promotes cell migration, proliferation, and permeability. VEGF activates eNOS through AKT phosphorylation, which in turn helps produce nitric oxide (NO) to regulate vasodilation and permeability^52,53^. Several studies highlight the significance of VEGF in wound healing in both *in-vitro* and *in-vivo* settings^48,54,55^. CD31 is highly expressed in endothelial cells and can be used to measure vessel density. 4-AP significantly increased the early post-burn expression of VEGF, eNOS, and CD31 at both the gene and protein levels on days 3 and 7. Our findings suggest that early 4-AP driven angiogenesis may attenuate inflammation through the transformation of M1 to M2 macrophages, and that this may ultimately accelerate burn healing through the transformation of keratinocytes and fibroblasts.

Burns kill epidermal-keratinocytes and dermal fibroblasts, along with hair follicle bulges through activation of inflammation, apoptosis, and necrosis^43^. We found that 4-AP markedly reduced apoptosis (TUNEL-positive cells) and significantly augmented cell proliferation (methyl green-positive cells) in both epidermal and dermal sites of burned skin tissues on days 3 and 7. This suggests an anti-apoptotic role for 4-AP. Burn-induced cell death occurs via the intrinsic pathway (involving BCL2 and related proteins) and the extrinsic pathway (involving death receptor signaling, e.g. TNFα interacting with TNF receptor 1)^44,45^, which both lead to the activation of caspase-9 and downstream effector caspase-3. Intrinsic apoptosis is initiated internally within the cell via BCL2 family proteins, including both pro-apoptotic (BAX and BAK) and anti-apoptotic (BCL2 and BCXL) proteins, all of which tightly regulate intrinsic mitochondrial-mediated apoptosis^46,47^. 4-AP treatment significantly reduced pro-apoptosis expression levels including BAX, caspase-9, and caspase-3 while increasing anti-apoptosis expression levels including BCL2 and BCLXL. These results support the anti-apoptosis function of 4-AP.

Keratinocytes are the dominant cells of the epidermis and they depend on a vascular network to interact with immune cells and fibroblasts to effect successful healing^56,57^. Specifically, M2 macrophages secrete several anti-inflammatory, anti-apoptotic, angiogenic, and other growth factors that act on keratinocytes^58^ to drive migration, proliferation, and differentiation during re-epithelialization^59,60^. The K10 marker is expressed in terminally differentiated keratinocytes and in hair follicle stem cells below the epidermis^26,27,63,64^, while K14 is associated with proliferative and migratory keratinocytes, as well as new hair follicle stem cells^61,62^. Our data showed that on days 7 and 21, the expression of both K10 and K14 was significantly higher in the epidermis and dermis in the 4-AP treatment group compared to the saline group. However, this increase was nonsignificant on day 3, suggesting that this effect of 4-AP treatment is consistent with late effects on burn healing.

After a burn, fibroblasts are recruited by activated to secrete ECM proteins^65,66^ by immune cells^31^secreting critical cytokines like FGF and TGFβ that play a role in the proliferation and differentiation of fibroblasts (vimentin-positive cells) into myofibroblasts (α-SMA-positive cells). The ECM, which makes up over 70% of the skin, includes fibrillar collagens (mainly collagen I (70%) and III (15%), fibronectin, proteoglycans, and other associated proteins^36,67^. Cytokines such as TNFα, interleukins, and growth factors such as TGFβ, VEGF, FGF, and EGF transcriptionally activate MMPs that control ECM protein degradation and synthesis. Excessive protease activity can lead to a counterproductive chronic healing response, so timed expression and activation of MMPs is essential for burn wound healing^68^. Among proteinases, MMP3 and MMP9 play a role in the degradation of ECM components such as collagen, fibronectin, and elastin following burn wounds, which support dermal repair and regeneration^69–71^. Our data align with these findings, showing that 4-AP significantly increased the expression of collagen III and I at both gene and protein levels as well as increasing MMP3 and MMP9 expression. This strongly supports a role for 4-AP in ECM repair and regeneration.

In conclusion, our study provides a rationale for a new therapeutic use of 4-AP in treating severe burns and promoting tissue regeneration. 4-AP helps to control inflammation, cell death, and the formation of new blood vessels, which in turn improves the healing of burn wounds by promoting the regrowth of skin and remodeling of underlying tissue. This research could pave the way for further exploration of how 4-AP affects the healing of burns, particularly by looking at the interactions between macrophages and other skin cells such as fibroblasts, endothelial cells, and keratinocytes, which are known to play roles in driving tissue regeneration.

## MATERIALS AND METHODS

### Vertebrate animals

Ten-week-old C57BL/6J male mice weighing 25 ± 3 g were procured from Jackson Laboratories (Bar Harbor, ME). All animal experiments were approved by the Institutional Animal Care and Use Committee (IACUC; protocol No. 2023 − 1071) at the University of Arizona College of Medicine in Tucson, AZ.

### Mouse model of skin burn injury

Mice were anesthetized by intraperitoneal injection of ketamine hydrochloride (100 mg/kg) and xylazine (10 mg/kg), purchased from Dechra Veterinary Products, KS, USA. The hair was depilated on the thoracolumbar dorsum region using a trimmer followed by hair removal cream (Nads). The skin was prepped for burn wound creation using a 70% alcohol swab (# 5110, Covidien) and 5% povidone-iodine applications (# NDC67618–155-16, Betadine). A full-thickness (third-degree) burn wound was created using a custom-made carbon rod that weighs 65 g (without external pressure) with a 10 mm surface area. The rod was heated to 95°C using temperature-controlled brass blocks and applied on the animal for 4 seconds (Fig. 1A). Following the burn wound creation, extended-release buprenorphine (3.25 mg/kg, # NDC86084–100-30, Ethiqa XR, Fidelis animal health) was given subcutaneously to all animals as an analgesic. The experimental animals (n = 6 animals/group) were randomly assigned to burn wounds (normal saline, 0.1 ml/mouse) and burn wounds with 4-AP (2 mg/kg; # A78403, Millipore Sigma) groups. 4-AP or saline was given intraperitoneally immediately after surgery and post-surgery days 1 to 21. The lowest starting dose of 4-AP in humans for multiple sclerosis is 10 mg once daily, and the calculated body mass-adjusted human equivalent dose of 4-AP in mice is 2 mg/kg^72,73^. All animals were euthanized using isoflurane anesthesia, followed by cervical dislocation on days 3, 7, and 21. Next, skin samples were harvested to analyze skin histomorphometry, apoptosis, angiogenesis, inflammation, and regeneration or remodeling using immunofluorescence (IF), qRT-PCR, and Western blotting.

### Measurement of burn wound closure

Burn wound healing was monitored daily, and burned images were taken using a digital camera on post-injury days 0, 3, 5, 7, 10, 14, 18, and 21 (Fig. 1A). The size of the burn wound areas was measured in pixels using NIH ImageJ-1.53e software (National Institutes of Health, Bethesda, MD, USA) using a reference scale. Wound closure is expressed as a percentage of day 0 wounds using the following formula.


$$\text{Wound}\;\text{closure}\left(\%\right)=\frac{\left(\text{Area}\;\text{of}\;\text{original}\;\text{wound}\;\text{at}\;\text{day}\;0-\text{Area}\;\text{of}\;\text{wound}\;\text{at}\;\text{postulated}\;\text{day}\right)}{\text{Area}\;\text{of}\;\text{original}\;\text{wound}\;\text{at}\;\text{day}\;0}\times 100$$

### Skin tissue processing and histological analysis

Skin tissue processing and hematoxylin and eosin (H & E) staining were performed as described in our previous publication^3^. Briefly, on days 3, 7, and 21, the skin was harvested from the wound bed using a 12 mm biopsy punch (# NC9253254, Fisher Scientific) and then halved at the center of the wound to use histology and gene or protein expression analysis. Next, skin tissues were fixed in 4% paraformaldehyde (# SC281692, ChemCruz) solution overnight, washed with 70% alcohol 3 times, and embedded in paraffin. The serial 5 μm thick vertical sections were taken from the skin tissues embedded blocks using a microtome (# HM315, GMI). Before staining, tissue sections were deparaffinized and serially rehydrated using xylene and alcohol respectively. The sections were stained with H&E staining kit as per the manufacture’s protocol with slight modifications (# ab245880, Sigma-Aldrich). Briefly, the tissues were stained with modified Mayer’s hematoxylin for 5 min and then incubated with a bluing reagent for 15 sec. Next, sections were stained with eosin (# 71204, Thermo Scientific) for 20 s, followed by dehydration with 95% and 100% alcohol for 5 min, two times. Sections were then cleared in xylene for 5 min, 2 times, and mounted with DPX mountant (# 06522, Sigma-Aldrich). H & E-stained slides were scanned using a slide scanner (MoticEasyScan, SF, USA) at 80 X magnification.

### Immunofluorescence staining and analysis

Skin tissue IF staining was performed as described in our previous publication^16^. Briefly, antigen retrieval was performed using a 10 mM sodium citrate buffer (pH 6.0) for 20 min at 95°C. Permeabilization and blocking of nonspecific binding were performed using 1% Triton X-100 and 5% goat serum respectively. Next, primary antibody staining was performed with CD31 (1:100, # 553370, BD Pharmingen), IL1β (1:250, # GTX74034, GeneTex), CD206 (1:400, # 141703, BioLegend), F4/80 (1:500, # SAB4501656, Sigma Aldrich), K10 (1:100, # SAB4501656, Sigma Aldrich), K14 (1:100, # NBP2–34270, Novus Biologicals), Vimentin (1:200, # 10366–1-AP, Thermo Fisher Scientific), and α-SMA (1:500, # 14–9760-82, Thermo Fisher Scientific) at 4°C overnight incubation. Then, samples were washed thrice with PBS and were incubated with the appropriate secondary antibody: Alexa Fluor 488 (1:1000, # A32723; Invitrogen) and Alexa Fluor 647 (1:1000, # A32733; Invitrogen) for 1h at room temperature. Staining without primary antibodies was used as a control for nonspecific fluorescence. Nuclei were counter-stained using ProLong^™^ Gold anti-fade reagent with DAPI (# P36935, Thermo Fisher Scientific) and sections were examined under a fluorescent microscope (# DM6000, Leica, IL, USA). The image analysis and quantification were performed using NIH ImageJ-1.53e software.

### TUNEL staining

To examine burned skin cell death or apoptosis, a terminal deoxynucleotidyl transferase dUTP nick end labeling (TUNEL) assay kit (HRP-DAB method) (# ab206386, Abcam) was used according to the manufacturer’s protocol. The process involved the following steps: 1. Deparaffinization of paraffin-embedded skin tissue sections using xylene, followed by serial rehydration in alcohol. 2. Treating with proteinase-K for 20 minutes at room temperature. 3. Washing with tris-buffered saline (TBS) and incubating in 30% H_2_O_2_ in methanol for 5 minutes at room temperature. 4. Washing with TBS buffer and incubating in terminal deoxynucleotidyl transferase (TdT) equilibration buffer for 30 minutes at room temperature. 5. Incubation in the TdT labeling reaction mixture for 90 minutes at 37°C in a humidified chamber. 6. Washing in a TBS buffer, addition of stop buffer, and incubation for 5 minutes at room temperature, followed by blocking buffer incubation for 10 minutes. 7. Wash off the blocking buffer and incubate the slides in 1X conjugate for 30 minutes at room temperature. 8. Subsequent washing with TBS, staining with 3,3’-diaminobenzidine (DAB) solution, and incubation at room temperature for 15 minutes. 9. Washing with distilled water to remove unbound DAB, then counter-staining the nuclei with methyl green and mounting with DPX mountant (# 0622, Sigma). Finally, slides were imaged using a slide scanner (MoticEasyScan, SF, USA) at 80 X magnification.

### RNA extraction and qRT-PCR analysis

Total RNA was extracted from the skin tissue using the RNeasy kit (# 74104, Qiagen). An equal amount of RNA (1000 ng) was reverse transcribed to cDNA using a high-capacity reverse transcription kit (# 4368814, Applied Biosystems). The primers (Invitrogen, Life Technologies) are detailed in the Supplement Table S1. qRT-PCR gene expression analysis was performed using Fast SYBR Green Master Mix (# 4367659, Applied Biosystems) with a Real-Time PCR System (Azure Cielo 6). The relative mRNA expression of the targeted genes was normalized against the glyceraldehyde 3-phosphate dehydrogenase (GAPDH) gene. The data were represented as fold change with 4-AP versus saline.

### Protein extraction and Western blotting analysis

Skin sample protein extraction was performed using T-PER^™^ extraction reagent (# 78510, Thermo Fisher Scientific). Briefly, the samples were homogenized at 4°C using 0.9–2.0 mm stainless steel beads (# SSB14B, Next Advance) at 5000 rpm for 5 min, followed by 13000 rpm for 20 minutes using a bullet blender (# BBX24, Next Advance Homogenizer). The extracted protein was quantified using a BCA assay kit (# 23225, Thermo Fisher Scientific). To identify targeted proteins, 20 to 40 μg of the isolated tissue protein samples were loaded and separated using 4–12% sodium dodecyl sulfate-polyacrylamide gel electrophoresis (# 4561044, GenScript). The gel was then transferred to a polyvinylidene fluoride (PVDF) membrane by wet transfer system (# L00686, GenScript) and blocked with 3% BSA in 1X TBST for 1 h at 37°C. The membrane was then incubated in the primary antibody overnight at 4°C. The primary antibodies used are mouse IL-1β (1: 500, # GTX74034, GeneTex), CD206 (1:400, # 141703, BioLegend), ARG-1 (1:2000, # 93668s, Cell Signaling), VEGF (1:1000, # JH121, Invitrogen), CD31, 1:1000, # 553370, BD Pharminogen), eNOS (1:2000, # 32027s, Cell Signaling), BAX (1:2000, # 14796, Cell Signaling), BCL2 (1:2000, # 4223, Cell Signaling), BCL-XL (1:2000, # 2764s, Cell Signaling), K10 (1:2000, # SAB4501656, Sigma Aldrich), K14 (1:5000, # NBP2–34270, Novus Biologicals), TGFβ (1:1000, # 3711s, Cell Signaling), Vimentin (1:5000, # 10366–1-AP, Thermo Fisher Scientific), α-SMA (1:500, # 14–9760-82, Thermo Fisher Scientific), and GAPDH (1:5000, # MA5–15738, Thermo Fisher Scientific). The membranes were then washed thrice with the tris buffered saline with Tween 20 (TBST) buffer and incubated with the respective secondary antibodies. The secondary antibodies used are an anti-rabbit HRP-linked antibody (# 7074, Cell signaling) and an anti-mouse HRP-linked antibody (# 7076, Cell signaling). The blots were developed using Super Signal West Pico PLUS chemiluminescent substrate ECL kit (# 34579, Thermo Fisher Scientific), and images were captured using a G-box ChemiXRQ gel imager. The bands were quantified using NIH ImageJ-1.53e software. All uncut original Western blotting images of targeted proteins are available in the supplementary materials.

### Collagen staining and analysis

The skin collagen staining was performed using Herovici’s collagen staining kit (# KTHER, StatLab). Briefly, the deparaffinized slides were immersed in Weigert’s hematoxylin for 5 minutes and then rinsed in running tap water for 45 seconds. Next, the tissues were stained with Herovici’s working solution for 2 minutes, followed by 1% acetic acid for 2 minutes. The slides were then dehydrated in absolute alcohol and cleared in xylene, each for 1 minute, three times. Finally, the slides were mounted using organic mountant (# 06522, Sigma Aldrich). The images were captured at 80 X magnification using a slide scanner (MoticEasyScan, SF, USA) and analyzed using NIH ImageJ-1.53e software.

### Statistical analysis

All data were analyzed using GraphPad Prism Version 10.1.1 (San Diego, USA). Comparisons between two or three groups with n ≥ 3 were performed using two-tailed, unpaired t-tests. All values are presented as mean ± SEM. Significance levels (P values < 0.05) were documented using standard symbols (*, **, and *** correspond to P < 0.05, P < 0.0021, and P < 0.0002, respectively).

## Data Availability

Data in the main text and the supplementary information are available from the corresponding authors upon reasonable request.
